# Cost-effectiveness of Human Papilloma Virus (HPV) vaccination in Nigeria: a decision analysis using pragmatic parameter estimates for cost and programme coverage

**DOI:** 10.1186/s12913-017-2758-2

**Published:** 2017-12-08

**Authors:** Obinna I. Ekwunife, Stefan K. Lhachimi

**Affiliations:** 1 Collaborative Research Group for Evidence-Based Public Health, Department of Prevention and Evaluation, Leibniz Institute for Prevention Research and Epidemiology – BIPS / University of Bremen, Bremen, Germany; 2 Department of Clinical Pharmacy and Pharmacy Management, Nnamdi Azikiwe University, Awka, Nigeria; 3 Institute for Public Health and Nursing Research – IPP, Health Sciences Bremen, University of Bremen, Bremen, Germany

**Keywords:** Human Papilloma Virus (HPV), Cervical cancer, Vaccine, Screening, Cost-effectiveness-analysis, Expected value of perfect information (EVPI), Nigeria

## Abstract

**Background:**

World Health Organisation recommends routine Human Papilloma Virus (HPV) vaccination for girls when its cost-effectiveness in the country or region has been duly considered. We therefore aimed to evaluate cost-effectiveness of HPV vaccination in Nigeria using pragmatic parameter estimates for cost and programme coverage, i.e. realistically achievable in the studied context.

**Methods:**

A microsimulation frame-work was used. The natural history for cervical cancer disease was remodelled from a previous Nigerian model-based study. Costing was based on health providers’ perspective. Disability adjusted life years attributable to cervical cancer mortality served as benefit estimate. Suitable policy option was obtained by calculating the incremental costs-effectiveness ratio. Probabilistic sensitivity analysis was used to assess parameter uncertainty. One-way sensitivity analysis was used to explore the robustness of the policy recommendation to key parameters alteration. Expected value of perfect information (EVPI) was calculated to determine the expected opportunity cost associated with choosing the optimal scenario or strategy at the maximum cost-effectiveness threshold.

**Results:**

Combination of the current scenario of opportunistic screening and national HPV vaccination programme (CS + NV) was the only cost-effective and robust policy option. However, CS + NV scenario was only cost-effective so far the unit cost of HPV vaccine did not exceed $5. EVPI analysis showed that it may be worthwhile to conduct additional research to inform the decision to adopt CS + NV.

**Conclusions:**

National HPV vaccination combined with opportunist cervical cancer screening is cost-effective in Nigeria. However, adoption of this strategy should depend on its relative efficiency when compared to other competing new vaccines and health interventions.

## Background

Cervical cancer is a major and growing public health challenge in Nigeria. The disease ranks as the second most frequent cancer among women in Nigeria [1]. Current estimates indicate that every year approximately 14,000 women are diagnosed with cervical cancer and 8240 die from the disease [1]. Cervical cancer deaths often occur in relatively young women between the ages of 45–50 years, who are raising children, caring for families, and contributing to communities [2, 3]. Without proper control, prevalence and burden of the disease will increase in the future especially as Nigerian population grows.

Cervical cancer is prevented by screening and treating women with cervical intra-epithelial neoplasia - CIN (i.e. abnormal cells found on the surface of the cervix). Screening technologies include cytology based screening, visual inspection with acetic acid (VIA), and the human papilloma virus (HPV) DNA test [4]. The disease can also be prevented by vaccinating girls between 9 to 13 years against HPV type 16 and 18 [4]. Currently, neither organized HPV screening nor national HPV vaccination programme exist in Nigeria. HPV screening is largely opportunistic and may reach a minority of the population who are not necessarily at the highest risk of disease [1].

World Health Organisation (WHO) recommends routine HPV vaccination for girls when the cost-effectiveness of vaccination strategies in the country or region has been duly considered [5]. In other words, national HPV vaccination programme could be added to cervical cancer screening programme if proven affordable. This is particularly so since prophylactic vaccination is not effective against infection from all oncogenic HPV types, and regular screening is still recommended among women that have received the vaccination [6]. Additionally, although the Vaccine Alliance (Gavi) and its partners provide poor countries with access to a sustainable supply of new and underused vaccines such as HPV vaccines for as little as US$ 4.50 per dose, national HPV vaccination in Nigeria still requires substantial set-up cost since adolescent vaccine delivery service is not in existence [7]. Therefore, a comprehensive cost-effectiveness analysis of HPV vaccination specific to Nigeria is important in order to offer appropriate policy advice.

Two older studies have assessed the cost-effectiveness of a vaccination-only strategy in Nigeria as part of broad analysis of several countries eligible for Gavi’s support [2, 8]. However, these studies did not consider cervical cancer screening as part of competing alternative strategy for Nigeria. Furthermore, these studies responded to data constraints typical in low and middle income countries (LMICs) by deriving a cost per vaccinated girl (CVG) estimate through a composite costing approach. Moreover, they did not report and justify the basis for the composite cost items. Another recent study assessed the most efficient combinations of HPV vaccination and screening coverage for the prevention of cervical cancer (CC) at different levels of expenditure [9]. The study however did not assess cost-effectiveness of HPV vaccination under realistically achievable conditions, but rather sought to calculate optimal coverage rate combination of both vaccination and screening. The study also considered cytology based screening rather than visual inspection with acetic acid (VIA) which is a low cost cervical cancer screening technology as currently recommended in LMICs such as Nigeria [4].

These shortcomings in the literature are not restricted to decision analytic models addressing the case of Nigeria. In general, scarcity, quality and accessibility of data have been identified as key challenges when conducting and using economic evaluations in many low and middle income countries (LMICs) [10]. In the particular case of economic evaluation of HPV vaccination in LMICs, crucial parameters—such as cost per vaccinated girl (CVG), screening coverage and vaccine coverage parameters—used in previous analysis are inevitably uncertain as there are no prior experience of HPV vaccination and cervical cancer screening in many of these countries [11]. For instance, the previous multi-country studies on economic evaluation of HPV vaccination in Gavi eligible countries assumed base case screening coverage parameters of 70%, which appears overly optimistic given what has been actually achieved by other LMICs under real-life conditions [2, 8]. South Africa has only achieved cervical cancer screening coverage of less than 20%, making the country with the highest coverage rate in the African region [12]. Some Latin American countries such as Costa Rica, Bolivia, El Salvador, Nicaragua, and Panama—usually considered economically and socially more advanced than most Sub-Saharan African countries—have screening coverage rates ranging from 10 to 20% [13].

Our study aimed to overcome those shortcomings by using pragmatic parameter estimates for cost and programme coverage when evaluating cost-effectiveness of HPV vaccination in Nigeria. Realistically achievable cost and programme coverage parameters were derived using the suggested recommendation for improved handling of LMIC-specific challenges in cost-effectiveness analysis of HPV vaccination [11]. The analysis was conducted in the light of different CC prevention scenarios including current scenario of opportunistic CC screening, national CC screening, national HPV vaccination and combination of both vaccination and screening. In the analysis, VIA was used as the CC screening option and two doses of HPV vaccine was considered [4]. Cost per vaccinated girl was adapted from a HPV vaccination demonstration project in Tanzania while treatment cost of HPV infection and sequelae were derived from a Nigerian health care setting. We accounted for differences in cost of HPV vaccination between urban and rural cities. South African national screening programme coverage rate was used as proxy for achievable screening coverage rate in Nigeria. The cost-effectiveness analysis specifically explored the following policy questions:

- Should Nigeria implement national HPV vaccination programme and national CC screening programme together or should national HPV vaccination programme be implemented only while CC screening remains opportunistic? Which strategy is the most cost-effective?
- Is national HPV vaccination programme (either with opportunistic screening or national CC screening) cost-effective if Nigeria is not able to purchase HPV vaccine at Gavi price? What is the least price for national HPV vaccination to remain cost-effective?
- Based on a single screening per lifetime as currently recommended, at what age will cervical cancer screening (combined with HPV vaccination) offer the most efficient outcome?
- What is the expected opportunity cost associated with choosing the most cost-effective strategy? In other words, should decision be made based on the result of our analysis or should additional research be conducted to inform perfect decision on cervical cancer prevention strategies?

## Methods

### Overview of competing strategies

The following cervical cancer prevention strategies were considered:

- Current scenario of opportunistic screening (CS) – The ‘current scenario’ depicts the existing situation in Nigeria where cervical cancer screening is opportunistic and funded ‘out of pocket’. Female population coverage rate of opportunistic screening is presently about 8.7% [1]. We assumed that opportunistic screening is once per life time for this analysis and that visual inspection with acetic acid (VIA) is used.
- National screening using VIA (NS): VIA involves a technique for examining the cervix with the naked eye, using a bright light source, after 1 min of 3–5% diluted acetic acid being applied using cotton swab or spray. The technique eliminates the need for cytologists and colposcopies. Detection of well defined aceto-white areas close to the squamoscolumnar junction indicates a positive test and this allows treatment with cryotherapy to be performed immediately. We assumed that screening is performed once for every woman in the target age of 30 years [4].
- National HPV vaccination in addition to opportunistic screening (CS + NV): This involves vaccination of 12 years preadolescent girls with two doses (0 and 6 months) of either of the two currently approved vaccines for prevention of high-risk HPV types 16 and 18 in Nigeria – Gardasil ® (Merck Sharpe and Dohme) and Cervarix® (GlaxoSmithKline) [4].
- National screening and national HPV vaccination (NS + NV): This involves HPV vaccination of 12 years preadolescent girls and screening (using VIA) of every woman in the target age of 30 years once in their lifetime.

### Disease model

**Fig. 1:**
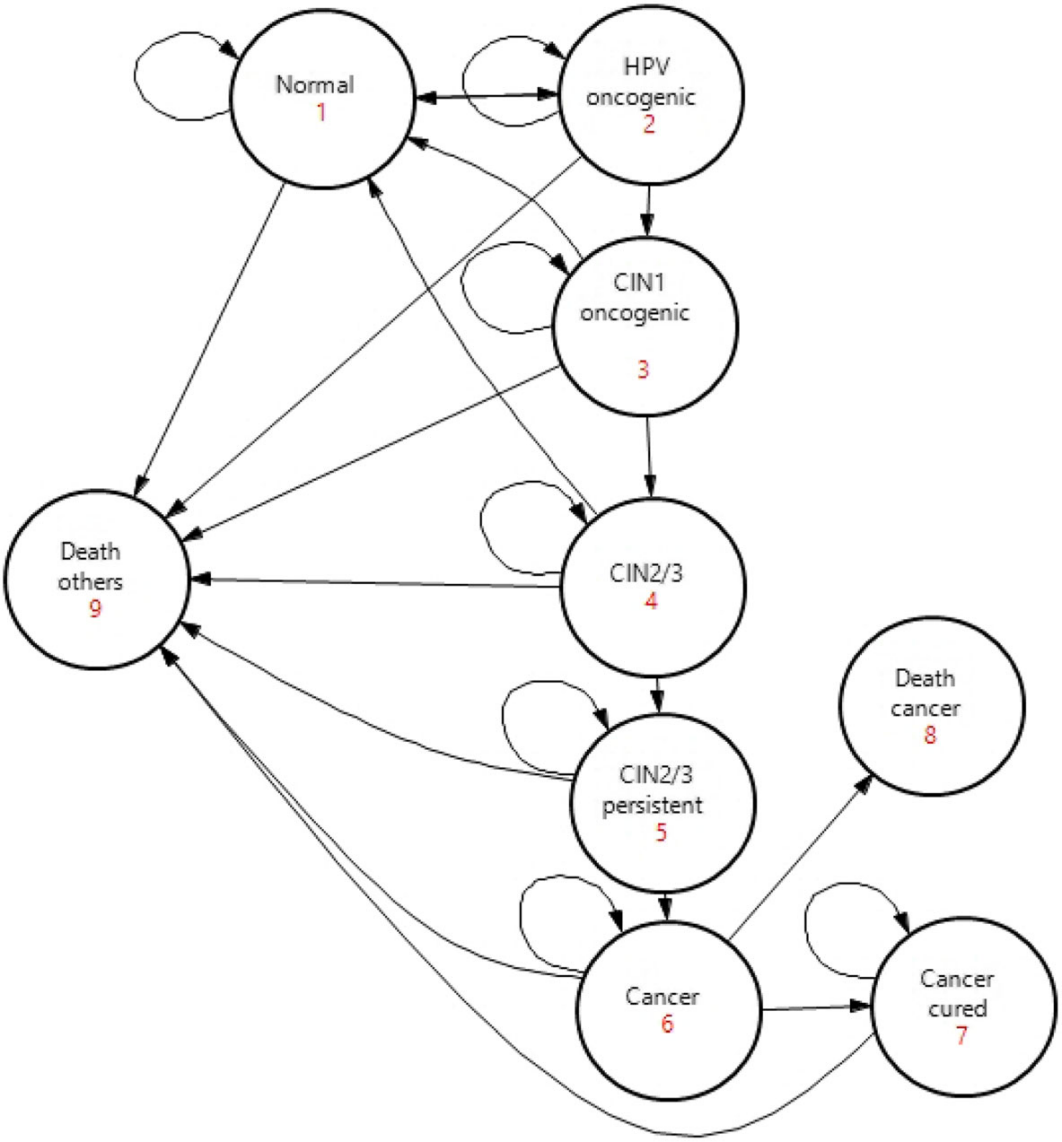
Cervical Cancer Disease Model. Disease model of cervical cancer disease is depicted. Circles correspond to the states and arrows represent the allowed transitions. HPV: Human papillomavirus; HPV: HPV infection type 16/18 and 10 most frequent non-vaccine HPV types 31/33/35/39/45/51/52/56/58/59 implicated in cervical cancer; CIN1: Cervical intraepithelial neoplasia, grade 1; CIN2/3: Cervical intraepithelial neoplasia, grade 2 and 3

The natural history for cervical cancer disease (Fig. 1) was remodelled from a previous Nigerian model-based study [9]. A patient level simulation (i.e. microsimulation frame-work) was adopted in our current study. The model followed one million women and was built using TreeAge Pro 2015® software. The model consisted of nine mutually exclusive health states. Each health state signifies different events which could occur throughout the natural history of HPV infection. The starting age of women in the model was 9 years, the age at which it is recommended for women to start HPV vaccination [14]. The terminal age was 99 years. The model ran in one-year-cycles. The transition probabilities between states were based on empirical data from the medical literature. Women with no infection could transit from a healthy state to a possible oncogenic HPV infection, which could regress over time to non-infected state, persist, or progress to cervical intraepithelial neoplasia 1 (CIN1). CIN1 could regress to non-infected state, persist, or progress to CIN2/3. CIN2/3 could persist or progress to persistent CIN2/3 grade. Persistent CIN2/3 grade could also persist or progress to cancer state (CC). Transition probability in some cases depended on age. Given the development of cancer, each individual could progress to death, or progress to a disease-free state. At each year, individuals were under an age-specific risk of death that is unrelated to cancer. Age-specific mortality rates were calculated from the Nigerian life table [15]. In the model, HPV vaccination prevented oncogenic HPV infection calculated based on estimated vaccine efficacy. Women detected of CIN1, CIN2/3 or persistent CIN2/3 grade through screening were treated with cryotherapy and all women with cervical cancer were treated.

### Model parameters

Model parameters regarding natural history of HPV 16/18 infections and diseases were obtained from available information on the natural history of HPV infection and cervical carcinogenesis based on the assumption that the natural history parameters are similar. However, Nigeria specific data were used whenever available. Specifically, the incidence of HPV infection in women was based on the calculation of the previous Nigerian based study [9]. Model parameters for demographics and clinical management of HPV-related diseases were also based on published Nigerian studies [9, 16, 17]. Following the previous Nigerian model-based study, vaccine efficacy was estimated as the weighted average vaccine efficacy for HPV types 16/18 and the 10 most frequent non-vaccine HPV types (HPV – 31/33/35/39/45/51/52/56/58/59) related to CC based on the clinical trial results of the AS04-adjuvanted HPV-16/18, with weights reflecting the relative frequency of the different HPV types in Nigeria women [9]. We assumed that HPV vaccine provides a lifelong protection as other related studies [18–20]. Details of parameters used in the model are shown in Table 1.

**Table 1:** Model input parameters

Parameters	Base Case [min – max], (α, β)		Distributions	Reference
Demographic & Clinical Parameters				
Rural population (%)	53%		–	[17]
Urban population (%)	47%		–	[17]
Vaccine and screening coverage and outcomes				
Vaccine efficacy for HPV 16/18	0.9 (124, 13.8)		Beta	[38]
HPV 16/18 prevalence in CC	66.9% (58.9% – 74.0%)		Triangular	[1]
Vaccine efficacy for 10 other HPV oncogenic types	0.302 (6.06, 14.02]		Beta	[39]
10 other HPV oncogenic type prevalence in CC	32.0% (14.9% – 72.9%)		Triangular	[1]
Assumed vaccine coverage^a^	66% [66% -70%]		Triangular	[40]
VIA sensitivity	70.8% [60% - 76%]		Triangular	[41–43]
Opportunistic screening coverage	8.7%		–	[1]
Assumed achievable screening coverage^b^	20%		–	[12]
Transition Probabilities				
	1 yr	99 yrs		
Age specific mortality rate	0.0098	0.3165	Table	[44]
	10 yrs	99 yrs		
Normal → HPV	0.14	0.00	Table	[9, 45]
HPV → CIN 1	0.05		–	[46]
CIN1 → CIN2/3	0.09		–	[47–49]
CIN2/3 → persistent CIN2/3	0.11		–	[48, 49]
	10 yrs	80 yrs		
HPV → Normal	0.29	0.55	Table	[46, 48–50]
CIN1 → Normal	0.45		–	[47–49, 51, 52]
CIN2/3 → Normal	0.23		–	[47–49, 51, 52]
Persistent CIN2/3 → Cancer	0.0–0.10		Uniform	[9]
Cancer → Cancer cured	0.084		–	[9]
Disability Weights				
Disability weight of cervical cancer state	0.08 [0.0–0.9]		Triangular	[29, 53]
Disability weight of death state	1		–	[29]
Disability weight of asymptomatic states	0		–	[29]
Cost data				
CVG at $13 per dose (urban/rural)	$35.16/$36.26		–	[24]
CVG at $11 per dose (urban/rural)	$31.16/$32.26		–	[24]
CVG at $9 per dose (urban/rural)	$27.16/$28.26		–	[24]
CVG at $7 per dose (urban/rural)	$23.16/$24.26		-	[24]
CVG at $5 per dose (urban/rural)	$19.16/$20.26		-	
CVG at $4.5 per dose (urban/rural)	$18.16/$19.26		–	[24]
Lifetime CC treatment cost	$1635.96		–	[9]
Screening cost (VIA)	$6.12 [3.95–25.26]		Triangular	[54]
Cryotherapy cost	$27.96 [14.57–337.68]		Triangular	[54]

*CVG* cost per fully vaccinated girl*,* VIA visual inspection with acetic acid*, CIN* cervical intraepithelial neoplasia*, CC* cervical cancer

^a^Coverage of 2nd dose of diphtheria toxoid, tetanus toxoid and pertussis vaccine (DTP2) used as a proxy to assess Nigeria’s capacity to introduce and implement the HPV vaccines

^b^Coverage of cervical cancer screening based on coverage rate achieved by South Africa

### Model validation

We assessed the predictive validity of the model by comparing its output and observed epidemiological data regarding cervical cancer incidence in Nigeria. Specifically, the model was run without any intervention and the CC incidence resulting from our model was compared to that reported by Globocan 2012 registry [21]. No intervention was applied for model validation since opportunistic screening prior to 2012 was unpopular in the country. CC incidence from our model was 17.5 /100,000 women per year compared to 17.1/100,000 women per year reported by Globocan 2012 registry [21].

### Cost estimate

Costing was based on health providers’ perspective. Cost items included were cost of treating CIN1, CIN2/3, as well as cost of screening and vaccination in a national screening or vaccination programme. Cost of opportunistic screening was excluded since it is paid ‘out of pocket’. Cost of treating CIN 1, CIN 2/3, and CC were obtained from the previous Nigerian model-based study [9]. The Nigerian model-based study (conducted in 2011) estimated the different treatment costs from a retrospective chart review performed at the University College Hospital at Ibadan, Nigeria. The chart review collected the medical resources used (outpatient health care professional visits, outpatient diagnostic procedures, outpatient treatment procedures, medications, and hospitalizations) to treat a patient with CIN1, CIN2/3 or CC. Resources used over a 1-year period were collected for ten patients with precancerous lesion while resources used for lifetime (from diagnosis until either death or cure) were collected for ten patients with CC. For our present study, we inflated the treatment cost of CIN1, CIN2/3 and CC from 2011 to 2015 US$ value. This was done by converting the treatment cost from 2011 US$ to naira equivalent, inflating to 2015 value using the consumer price index, and then converting back to US$. We used exchange rates published by Central bank of Nigeria [22] and consumer price index published by World Bank [23]. Costs of cervical cancer screening with VIA and cryotherapy were obtained from a Ghanaian study [24] and were inflated to 2015 US$ value using a web-based CPI inflation calculator [25]. Future costs were discounted at 3%. Details of treatment cost of CIN1, CIN2/3 and CC are shown in Table 1.

Cost per vaccinated girl (CVG) was estimated by adjusting cost of HPV vaccination delivery in Tanzania to the Nigerian setting as described in another study [26]. In particular, the cost adjustment was achieved by modifying cost items–social mobilization/information, training, procurement (except for vaccine cost), vaccination, cold storage, and administration/supervision–based on the difference in local purchasing power between Tanzania and Nigeria. Difference in local purchasing power was computed with a web-based cost of living calculator [27]. In accordance with recent recommendations, vaccine procurement cost was modified to reflect the cost of two doses at $4.50 per dose instead of three doses at $5 as per original study [14]. Vaccine price of US$ 4.50 reflects the price being offered by Vaccine Alliance (Gavi) for countries eligible for support, while vaccine price of US$ 13 represents the lowest public sector price offered by HPV vaccine manufacturers [28]. Except for the vaccine cost, all other vaccine delivery associated costs were inflated from 2012 (i.e. the year of publication) to 2015 US$ value. Details of CVG are shown in Table 2.

**Table 2:** Economic Costs per Fully-immunized Girl (US$) in a Scaled-up Regional School based HPV vaccination Programme

Cost items	Source Data (Mwanza Vaccine Project, Tanzania) [21]		Gavi vaccine price (US$ 4.5) [23]		Lowest public sector price (US$ 13) [23]	
	Urban	Rural	Urban	Rural	Urban	Rural
Social Mobilization/IEC	0.5	0.5	0.44	0.44	0.44	0.44
Training	0.3	0.5	0.26	0.44	0.26	0.44
Procurement^a^	18.7	19.4	12.46	13.11	29.46	30.11
Vaccination	5.0	4.4	4.39	3.87	4.39	3.87
Cold Storage	0.2	0.3	0.18	0.26	0.18	0.26
Waste Management	0.0	0.0	0.0	0.0	0.0	0.0
Admin/Supervision	0.5	1.3	0.44	1.14	0.44	1.14
Total	25.3	26.6	18.16	19.26	35.16	36.26

^a^Vaccine procurement cost was modified to reflect the cost of two doses at US$4.5 per dose instead of three doses at US$5 as per original study

### Benefit estimate

Disability adjusted life years (DALYs) attributable to cervical cancer mortality was calculated by tracking years of life lost due to cervical cancer death (YLL), and years lived with disability due to cervical cancer (YLD) through the model [29]. DALY was calculated by combining years lived with disability (YLD) and years of life lost (YLL) for each yearly cycle. YLD was calculated as follows: YLD = Number of cases × duration till remission or death × disability weight. YLL was calculated as follows: YLL = Number of deaths due to cervical cancer × life expectancy at the age of death [30]. Disability weights from global burden of disease study were used to calculate years lived with disability [29]. DALY estimate were discounted at 3%.

### Analysis

We identified the suitable cervical cancer prevention policy in Nigeria by calculating the incremental costs-effectiveness ratio (ICER). ICER represents the average incremental cost associated with one additional DALY averted. The traditional 1–3 GDP per capita proposed by Commission on Macroeconomics and Health of the World Health Organization was used as cost-effectiveness threshold [31]. Thus, a strategy with ICER below the Nigerian GDP per capita (US$ 3203.3) was judged very cost-effective and a strategy with ICER below three times Nigerian GDP per capita (approximately US$ 9610) was judged cost-effective [32].

Probabilistic sensitivity analysis (PSA) approach was used to assess parameter uncertainty. The PSA allowed exploration of the joint uncertainty in costs and effects across natural history parameters, screening and vaccination parameters and costs. The distributions for PSA are summarized in Table 1. In addition to probabilistic sensitivity analysis, one-way sensitivity analysis was used to explore the robustness of the policy recommendations to key parameters alteration. Specifically the impact of greater screening intensity, higher screening coverage, and higher or lower vaccination coverage on policy recommendation were explored using one-way sensitivity analysis.

The expected value of perfect information (EVPI) was calculated to determine the expected opportunity loss surrounding the scenario recommended as the most cost-effective. Non parametric approach was used to calculate population EVPI [33]. The population EVPI was conducted for one million women over the life cycle of 91 years. EVPI was calculated from a simulated output of a million iterations generating net-benefit for each of the four competing scenarios. Average maximum net benefit from all the iterations was subtracted from average net-benefit of the most cost-effective scenario (CS + NV). The result was multiplied by discounted effective population in order to obtain the value of perfect information for different willingness-to-pay thresholds. One hundred draws from the probability distribution (2nd order Monte Carlo simulation) to calculate discounted effective population across different cost-effectiveness thresholds was conducted. A discount rate of 3% was used. EVPI was calculated using Microsoft Excel, 2010.

## Result

### Policy recommendation

The outcomes of the cost-effectiveness analysis (CEA) for the first three policy questions are shown in Table 3. The first policy question assessed whether a national HPV vaccination programme should be added to the current scenario of opportunistic cervical cancer screening (CS + NV) or to a national screening programme (NS + NV). The results of the CEA showed that CS + NV option was the only cost-effective option. National vaccination programme added to opportunistic screening (CS + NV) resulted in an ICER of $7930/DALY averted which was less than the cost-effectiveness threshold of $9610. CS + NV scenario also reduced cervical cancer incidence by 34%. National cervical cancer screening and national HPV vaccination (NS + NV) and national screening programme alone was not cost-effective.

**Table 3:** Results of base case cost-effectiveness of analysis

Name of strategy	Costs (US$)	Effectiveness (DALYs)	Incremental Costs	Incremental Effectiveness (DALY averted)	ICER (US$ / DALY averted)	Annual CC incidence per 100,000	Remark
Gavi vaccine price ($4.5/dose)							
CS	8.00	0.00427	–	–	–	17.08	–
NS alone	10.27	0.00418	2.27	0.00009	–	16.52	Dom
CS + NV	18.23	0.00298	10.23	0.00129	7930	11.52	R
NS + NV	20.16	0.00292	1.93	0.00006	32,167	11.16	NR
Lowest vaccine price offered to public sector ($13/dose)							
CS	8.05	0.00431	–	–	–	17.09	–
NS alone	10.31	0.00421	2.26	0.00010	–	16.53	Dom
CS + NV	29.76	0.00305	21.71	0.00126	17,230	11.68	NR
NS + NV	31.71	0.00299	1.95	0.00006	32,500	11.34	NR
Non Gavi vaccine price (CS + NV)							
$11/dose	27.02	0.00300	19.00	0.00128	14,845	11.64	NR
$9/dose	24.25	0.00297	16.22	0.00133	12,202	11.52	NR
$7/dose	21.58	0.00298	13.45	0.00136	9900	11.52	NR
$5/dose	18.90	0.00301	10.72	0.00136	7897	11.57	R
Most efficient cervical cancer screening age (CS + NV)							
30 years	18.23	0.00298	10.23	0.00129	7933	11.52	R
40 years	18.29	0.00303	10.20	0.00128	7947	11.79	R
50 years	19.37	0.00306	10.26	0.00127	8116	11.73	R

Cost-effectiveness threshold = US$ 9609.9/DALY averted

$$ \mathrm{ICER}=\frac{Strategy\kern0.5em \cos t\hbox{-} Current\kern0.5em scenario\kern0.5em \cos t}{Current\kern0.5em scenario\kern0.5em DALYs\kern0.5em lost\hbox{-} Strategy\kern0.5em DALYs\kern0.5em lost} $$

*CS* current scenario of opportunistic screening, *NS* National cervical cancer screening, *NV* National HPV vaccination, *DALY* disability adjusted life years, *CC* cervical cancer, *R* recommended, *NR* not recommended, *Dom* Dominated

The second policy question assessed the significance of purchasing HPV vaccines at non Gavi price. Vaccine price of $13 was applied since this was the lowest price the vaccine manufacturers have offered the vaccine to the public sector. The result of the CEA showed that CS + NV scenario will not be cost-effective if vaccines are purchased at $13. The ICER under this scenario increased from $7930/DALY averted at vaccine price of $4.5/dose to $17,230/DALY averted at vaccine price of $13/dose. Furthermore, CS + NV scenario is only cost-effective if HPV vaccine is purchased at not more than $5/dose. The ICER under vaccine price of $5/dose for CS + NV scenario was $7897/DALY averted.

The third policy question aimed to identify the most cost-effective age for screening. Based on one screening per lifetime, screening at age 30 was the most efficient screening age. Screening at age 30 resulted in the least ICER of $7933/DALY averted for current scenario plus national vaccine (CS + NV).

### Robustness of policy recommendation

One-way sensitivity analysis was used to explore the robustness of the option that emerged cost-effective i.e. current scenario plus national vaccination (CS + NV). CS + NV scenario remained robust in spite of parameter alterations as shown in Table 4. Specifically, ICER of CS + NV remained below cost-effectiveness threshold with more screening frequency (twice and thrice per lifetime), at higher screening coverage rates (40 and 60%), and at higher or lower vaccination coverage rates (50 or 90% respectively).

**Table 4:** Result of sensitivity analysis

Name of strategy	Costs (US$)	Effectiveness (DALYs)	Incremental Costs	Incremental Effectiveness (DALY averted)	ICER (US$ / DALY averted)	Annual CC incidence per 100,000	Remark
2 × screening per life time							
CS	8.05	0.00429	–	–	–	17.06	–
NS alone	12.06	0.00414	4.01	0.00015	–	16.03	Dom
CS + NV	18.32	0.00302	10.27	0.00127	8087	11.66	R
NS + NV	21.84	0.00293	3.52	0.00009	39,111	11.03	NR
3 × screening per life time							
CS	8.07	0.00430	–	–	–	17.03	–
NS alone	13.58	0.00413	5.51	0.00017	–	15.85	Dom
CS + NV	18.26	0.00300	10.19	0.00130	7838	11.61	R
NS + NV	23.31	0.00288	5.05	0.00012	42,083	10.80	NR
National screening programme achieving 40% coverage							
CS	8.02	0.00427	–	–	–	16.95	–
NS alone	13.48	0.00402	5.46	0.00025	–	15.52	Dom
CS + NV	18.16	0.00295	10.14	0.00132	7682	11.38	R
NS + NV	23.00	0.00280	4.84	0.00015	32,267	10.49	NR
National screening programme achieving 60% coverage							
CS	7.98	0.00427	–	–	–	17.15	–
NS alone	16.61	0.00387	8.63	0.00040	–	14.69	Dom
CS + CV	18.26	0.00301	10.28	0.00126	8159	11.59	R
NS + NV	25.98	0.00276	7.72	0.00025	30,880	10.10	NR
National vaccination programme achieving 90% coverage							
CS	8.02	0.00429	–	–	–	16.90	–
NS alone	10.30	0.00420	2.28	0.00009	–	16.39	Dom
CS + NV	21.70	0.00257	13.68	0.00172	7953	9.73	R
NS + NV	23.53	0.00253	1.83	0.00004	45,750	9.46	NR
National vaccination programme achieving 50% coverage							
CS	8.00	0.00426	–	–	–	17.02	–
NS alone	10.28	0.00417	2.28	0.00009	–	16.47	Dom
CS + NV	15.65	0.00336	7.65	0.00090	8500	12.87	R
NS + NV	17.67	0.00329	2.02	0.00007	28,857	12.48	NR

Cost-effectiveness threshold = US$ 9609.9/DALY averted

$$ ICER=\frac{Strategy\kern0.5em \cos t- Current\kern0.5em scenario\kern0.5em \cos t}{Current\kern0.5em scenario\kern0.5em DALYs\kern0.5em lost- Startegy\kern0.5em DALYs\kern0.5em lost} $$

Gavi vaccine price ($4.5/dose) used for all analysis

*CS* current scenario of opportunistic screening

NS, National cervical cancer screening, *NV* National HPV vaccination, *DALY* disability adjusted life years, *CC* cervical cancer, *R* recommended, *NR* not recommended, *Dom* dominated

### Population expected value of perfect information

**Fig. 2:**
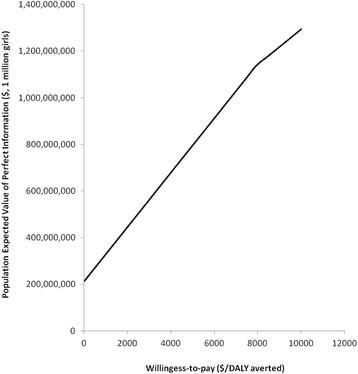
Population expected value of perfect information

Expected value of perfect information (EVPI) was calculated to determine the expected opportunity loss surrounding the recommendation of current scenario plus national vaccination (CS + NV) as the most cost-effective option. In other words, EVPI assessed the maximum expected value that could be spent on additional research so as to obtain perfect information to inform the policy decision of selecting CS + NV as a cost-effective option. For a population of one million girls, the EVPI of the most cost-effective scenario (CS + NV) was $1,138,060,192. In other words, a maximum of about $1.1 billion could be spent on additional research to obtain perfect information in order to inform the decision to adopt CS + NV (Fig. 2).

## Discussion

This analysis employed a microsimulation model to assess cost-effectiveness of HPV vaccination in Nigeria under different policy scenarios. The cost-effectiveness analysis was conducted using realistically achievable cost and programme coverage parameters. Robustness of the recommended policy scenarios were assessed using one-way sensitivity analysis. The expected value of perfect information (EVPI) was used to assess the maximum expected value that could be spent on additional research so as to obtain perfect information to inform policy recommendation. The result showed that combination of the current scenario of opportunistic screening and national HPV vaccination programme (CS + NV) was the only cost-effective option. It was also a robust policy option as it remained cost-effective in spite of changes in screening and vaccination coverage as well as screening intensity. However, combination of the current scenario of opportunistic screening and national HPV vaccination programme (CS + NV) is only cost-effective so far the unit cost of HPV vaccine does not exceed $5. Furthermore, greatest efficiency is achieved when women undergo cervical cancer screening at the age of 30 rather than later. EVPI analysis showed that it may be worthwhile to conduct additional research to inform the decision to adopt CS + NV. This is because the expected opportunity loss surrounding the adoption of CS + NV is much (approximately $1.14 billion for a population of one million girls).

The result of our analysis is of significance for decision on cervical cancer prevention strategies in Nigeria. The result shows that HPV vaccination added to opportunistic cervical cancer screening (CS + NV) is cost-effective. However, it is important to add that it is not very cost-effective as its ICER is higher than Nigerian GDP/capita of $3203.3 [32]. The ICER of $7930/DALY averted shows that the CS + NV option may not rank top in the priority list of new health interventions to be adopted in Nigeria even though it is cost-effective. Furthermore, a recent study suggested an alternative 0.51 times GDP per capita as threshold for determining cost-effective interventions [34]. Applying this conservative cost-effectiveness threshold will result in CS + NV judged as not cost-effective. Therefore adoption of HPV vaccination should depend on how cost-effective it is when compared to other competing newly available vaccines and health interventions. Comparative cost-effectiveness analysis of these competing vaccines and health intervention is needed to provide clear insights to decision makers.

To ensure sustainability of cervical cancer prevention programme in Nigeria, we recommend ‘out-of-pocket’ payment for cervical cancer screening. This is particularly so since it is debatable whether Nigeria (like other developing countries) could achieve a high coverage of well-organized and high-quality cervical cancer screening. An effective nationally organized screening programme requires a number of assets including a well-organized call-recall system, an accurate register of women and their screening history, adequate follow-up and treatment for screen positive women, rigorous quality control to ensure good test accuracy and good screening coverage rates across the eligible population. These may be difficult and costly to realize in Nigeria given the existing weak health infrastructure. In our view, adoption of a national HPV vaccination and opportunistic cervical cancer screening should be combined with an awareness programme to sensitize women on the necessity for cervical cancer screening, so as to ensure high demand for screening.

We conducted an expected value of perfect information analysis to further explore the expected cost of uncertainty surrounding the cost-effective option (CS + NV). Since the objective of this analysis is to maximize health expenditure given the Nigerian health budget constraint, the EVPI represents the maximum amount that Nigerian health policy makers should be willing to pay for additional evidence to inform perfect decision on cervical cancer prevention strategies. In cases where EVPI is too large, it indicates that the amount which could be forgone is too large and thus it may be better to conduct additional research to inform decision. The EVPI of adopting national HPV vaccination in combination with opportunistic screening was very high. The EVPI result further lends support to the fact that although HPV vaccination is cost-effective, there are lots of uncertainties surrounding its recommendation as a cost-effective strategy. Thus it may be worthwhile to resolve these uncertainties through additional research. For instance, a demonstration project to establish the actual cost of vaccination, actual cost of screening and cryotherapy treatment, and uptake rate of both vaccination and screening will allow for better decisions.

The result of our analysis is comparable to what has been reported in literature. Firstly, only cervical cancer screening has been shown to be less efficient compared to a combination of screening and vaccination. Sinanovic et al. showed that cervical cancer screening only strategy is dominated by screening combined with vaccination strategy in South Africa [35]. As reported by another study, cervical cancer screening only strategy was also less efficient and was dominated in both South Africa and Uganda [8]. Secondly, $5 per dose has been shown to be an optimal HPV vaccine cost for developing nations. For instance, similar analysis conducted for Extended Middle East and North Africa (EMENA) found that HPV vaccination was cost-effective in all but five countries at a cost per vaccinated girl of I$25 ($5 per dose) [36]. Lastly, two older multi-country analyses have shown that HPV vaccination could be very cost-effective in Nigeria [2, 8]. Goldie et al. reported an ICER for HPV vaccination of I$1080/DALY averted at I$5/dose (I$25 per vaccinated girl) [2]. Kim et al. reported an ICER for HPV vaccination of I$300/DALY averted at I$5/dose (I$25 per vaccinated girl) [8]. In our analysis however, ICER of HPV vaccination was higher for HPV vaccination ($7930/DALY averted) at $4.5/dose. The difference in the ICERs is because the older studies appear to yield a much greater estimated health benefit of vaccination. The DALYs averted per vaccinated girl by Goldie’s study is over ten times higher than the reduction in DALYs reported by our study. The older studies make many simplifying assumptions and were developed to assess the cost-effectiveness of HPV vaccination in multi-countries. Our model was based on a previous one developed specifically for Nigeria, developed with some country specific epidemiologic parameters. Suffice to state that the difference in base case reduction of cervical cancer incidence between our study and the original model (i.e. by Demarteau et al.) was because we applied more realistic parameters (cervical screening coverage of 20% and HPV vaccination coverage of about 67%) while the original model applied optimistic parameters (cervical screening coverage and HPV vaccination coverage of 100%).

There are several limitations in our study which has to be taken into consideration when interpreting the result of the analysis. Our analysis used static modelling which does not capture the impact of herd immunity. Thus, the indirect protection offered by HPV vaccination is not captured, which may underestimate the effect of vaccination. For instance, one study showed that at coverage levels between 50 and 70%, indirect effect of vaccination accounted for an additional 10% cancer reduction compared to the mean projected estimate in the base case [37]. We also did not consider the impact of vaccination on other HPV-related diseases that are attributable to HPV 16/18–including anal cancer, vulvar and vaginal cancer, and oropharyngeal and oral cancer–and thus may have underestimated potential benefits of the vaccine. Secondly, some of the key parameters like cost per vaccinated girl and screening coverage were derived from other country’s experience. This in reality may not obtain in Nigeria. Additionally, the cost of initiating screening programme at the national level was not included in the analysis and this could also influence the feasibility of its introduction. Lastly, the specificity of VIA was not considered in the model although it is unlikely that the conclusions of the cost-effectiveness analysis will change, since screening with VIA was dominated. Despite these limitations, we applied the most conservative estimates given the available data and experience to provide initial insight to policy makers and potential payers in Nigeria. Country implementation of HPV vaccination will require a second series of decisions and corresponding new analyses that will also consider the likelihood of uptake and acceptability.

## Conclusion

In summary, our analysis showed that HPV vaccination added to the current scenario of opportunistic screening is cost-effective. Decision to adopt HPV vaccination in Nigeria should depend on its relative efficiency when compared to other competing new vaccines (e.g. rotavirus vaccines) and other health interventions (e.g. neglected and non-communicable disease interventions). To this effect, comparative cost-effectiveness analysis of these competing vaccines and health intervention is needed to provide clear insights to decision makers. Such analysis will establish a priority list which will aid decisions on interventions to adopt, especially with given limited health budget.

## Abbreviations

CC: Cervical cancer
CEA: Cost-effectiveness analysis
CIN: Cervical intra-epithelial neoplasia
CS: Current scenario of opportunistic screening
CVG: Cost per vaccinated girl
DALY: Disability adjusted life years
EMENA: Extended Middle East and North Africa
EVPI: Expected value of perfect information
GDP: Gross domestic product
HPV: Human Papilloma Virus
ICER: Incremental cost-effectiveness ratio
LMIC: Low and middle income countries
NS: National screening
NV: National vaccination
PSA: Probabilistic sensitivity analysis
VIA: Visual inspection with Acetic acid
WHO: World Health Organization
YLD: Years lived with disability
YLL: Years of life lost
